# The Interplay between Dysregulated Ion Transport and Mitochondrial Architecture as a Dangerous Liaison in Cancer

**DOI:** 10.3390/ijms22105209

**Published:** 2021-05-14

**Authors:** Stine F. Pedersen, Mette Flinck, Luis A. Pardo

**Affiliations:** 1 Department of Biology, Faculty of Science, University of Copenhagen, 2100 Copenhagen, Denmark; mette.flinck@bio.ku.dk; 2 Oncophysiology Group, Max Planck Institute for Experimental Medicine, 37075 Göttingen, Germany

**Keywords:** mitochondrial fission, mitochondrial fusion, calcium, pH, potassium, membrane potential, metabolism, apoptosis, cell cycle, metastasis

## Abstract

Transport of ions and nutrients is a core mitochondrial function, without which there would be no mitochondrial metabolism and ATP production. Both ion homeostasis and mitochondrial phenotype undergo pervasive changes during cancer development, and both play key roles in driving the malignancy. However, the link between these events has been largely ignored. This review comprehensively summarizes and critically discusses the role of the reciprocal relationship between ion transport and mitochondria in crucial cellular functions, including metabolism, signaling, and cell fate decisions. We focus on Ca^2+^, H^+^, and K^+^, which play essential and highly interconnected roles in mitochondrial function and are profoundly dysregulated in cancer. We describe the transport and roles of these ions in normal mitochondria, summarize the changes occurring during cancer development, and discuss how they might impact tumorigenesis.

## 1. Introduction

Since the first observation of mitochondria in the 1840s, a century had to pass until it was evident that active respiration occurs in the organelle [1]. It still took decades until the community started looking at mitochondria as a very complex system playing central roles in adapting cells to changes in the environment and energetic needs. Even the plural “mitochondria” seems misleading in this context because it implies a collection of independent entities. In contrast, mitochondria form a dynamic, interconnected network occupying a significant fraction of the cell volume. Mitochondria undergo constant morphological changes and respond to a plethora of cellular and environmental signals. They participate in the integration and modulation of the response to such signals, not only through adapting energy production and consumption, but also by playing key roles in signaling, cell cycle progression, cell motility, cell fate decisions, and by coordinating adaptive strategies with other cellular structures. As such, they engage in direct interactions with the endoplasmic reticulum (ER), trans-Golgi network, lysosomes, lipid droplets, peroxisomes, and the plasma membrane, and are embedded in—and interact with—a dynamically regulated cytoskeletal network [2,3]. Therefore, it is not surprising that the pervasive phenotypical changes occurring during cancer development profoundly impact mitochondrial architecture and function and that this, in turn, plays a key role in the cancer cell phenotype. The relation between mitochondria and cancer has been the topic of several excellent reviews (e.g., [4,5]). However, even though transport of ions and metabolites is a core mitochondrial function without which there could be no ATP generation, the link between mitochondria and ion homeostasis in cancer has been largely ignored. This is particularly important because of the profound dysregulation of ion homeostasis that characterizes both the tumor microenvironment and the cancer cells themselves (e.g., [6,7,8,9,10,11]). Although the importance of ion transport mechanisms for mitochondria and of mitochondria and ion transport for cancer progression are well established, translating this into safe and efficacious therapeutic approaches requires an integrated understanding of the processes. 

**Figure 1:**
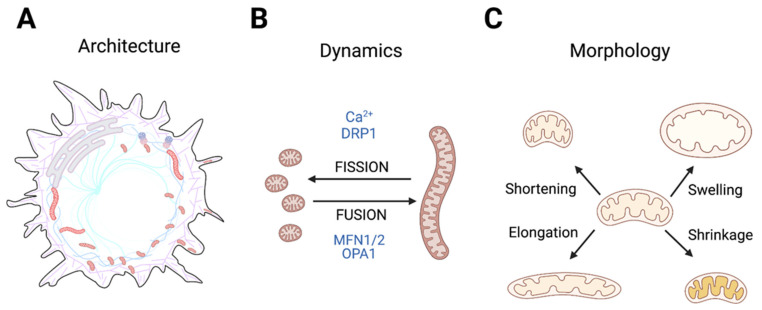
Schematic representation of the concepts of mitochondrial architecture (**A**), mitochondrial dynamics (**B**), and morphology (**C**) as used through this review. (**A**) Mitochondrial architecture refers to the positioning and interactions of the mitochondrial network and individual mitochondria with other cellular structures, such as the cytoskeleton and other membranes. (**B**) Mitochondrial dynamics encompasses the processes of fusion and fission. Finally, mitochondria morphology (**C**) refers to swelling, shrinking, elongation, and cristae reorganization, without fusion or fission events. All figures in this review have been created using BioRender.com.

This review aims to provide such an integrative view of the reciprocal relationship between ion transport and mitochondria and how it can contribute to cancer cell phenotypes. We limit our discussion to Ca^2+^, H^+^, and K^+^ ions and their associated channels and transporters, which are widely dysregulated in cancer (e.g., [9,10,11]) and of particular interest for mitochondrial morphology and function. We briefly review the mechanisms and roles of Ca^2+^, H^+^, and K^+^ transport in mitochondria and the evidence linking altered homeostasis of these ions in tumors to changes in key mitochondrial functions. Given the numerous excellent reviews of mitochondrial metabolism in cancer available elsewhere (e.g., [5,12], we will focus here on mitochondrial architecture and its important roles in responses to nutrient stress, signaling, and cell fate decisions, including cell cycle progression, stemness, and cell death. Throughout the text, we will use the term mitochondrial architecture to refer to the combination of the complexity, positioning, and tethering of the mitochondrial network to other structures (Figure 1A); mitochondrial dynamics will be used to describe fusion-fission events (Figure 1B), and finally, mitochondrial morphology will refer to more localized changes, such as swelling or cristae reorganization, which might or might not affect the network extensively (Figure 1C). 

## 2. Interdependent Ion Fluxes Drive Mitochondrial Function

The structure of the mitochondria, harboring two membranes with very different permeation properties separated by an intermembrane space (IMS) of variable size and organization, is unique in terms of ion transport. It is traditionally assumed that the ion concentrations in the cytoplasm and the IMS are identical due to the high permeability of the outer mitochondrial membrane (OMM). Although this is likely not entirely correct—local ion concentrations—e.g., along cristae—likely differ, and the permeability of the OMM can be modulated (see Section 2.1); we will also here assume equal ion concentrations on both sides of the OMM. 

**Figure 2:**
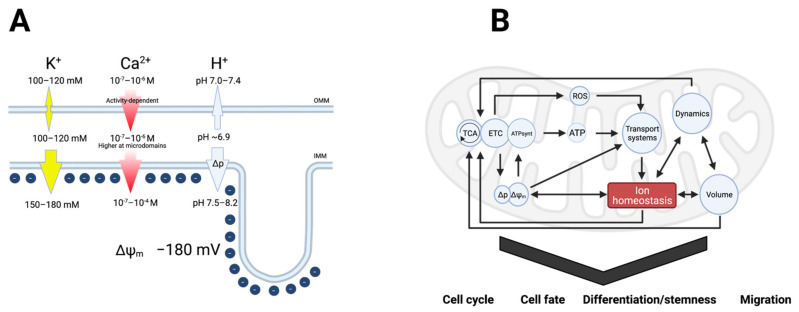
(**A**) Major electrochemical gradients for ions in the mitochondria. Chloride and larger organic ions have been ignored for clarity. The direction of the arrows indicates that of the concentration gradient, and the size of the arrow is roughly proportional to its magnitude. (**B**) Schematic representation of the reciprocal interactions between ion gradients and all aspects of mitochondrial physiology. Transport of Ca^2+^, H^+^, and K^+^ depend on Δψ_m_, as ATP synthesis does. ATP modulates the permeability of some channels and is required for active transport and the maintenance of gradients. K^+^ transport is crucial for determining mitochondrial volume, which affects Δψ_m_ and ATP synthesis.

The electrochemical gradients for ion transport across the inner mitochondrial membrane (IMM) play a pivotal role in all aspects of mitochondrial physiology. In other words, mitochondrial function relies fully on ion gradients and ion transport. These are interdependent since gradients are generated by ion transport, and ion transport depends on both the inner mitochondrial membrane potential (Δψ_m_, about −180 mV in healthy mitochondria) and the ion concentrations in the mitochondrial matrix and the cytosol (Figure 2A). Depending on the ionic species, the gradients can be minimal (like for K^+^, which is less than twice more abundant in the matrix than in the cytoplasm [13,14,15]) or very large (orders of magnitude for Ca^2+^ and H^+^). In turn, mitochondrial processes impact not only Δψ_m_ but also the free cytosolic Ca^2+^ ([Ca^2+^]_i_) and H^+^ concentrations. Finally, osmotic water flux driven by the ion fluxes (K^+^ in particular) between the cytosol and mitochondria modulates mitochondrial matrix volume [16] (Figure 2B). 

The discovery of ion channels in the IMM was an unexpected outcome of patch-clamp measurements in mitoplasts (mitochondria devoid of the OMM) in the 1980s [17]. It is now known that abundant K^+^ channels (voltage-gated, Ca^2+^-activated, ATP-dependent, Na^+^-activated [18], and two-pore K^+^ channels), as well as Ca^2+^, Na^+^-, and non-selective cation channels and anion channels participate in ion flows across the IMM [19]. The roles of the channels may not be limited to canonical ion transport functions, as in some cases direct interactions of particular ion channels, with, for example, components of the electron transport chain (ETC) [20] or the apoptosis machinery [21] have been described. When comparing the topology of mitochondrial channels to that of plasma membrane-localized channels, there is a tendency to consider the matrix “intracellular” and IMS “extracellular.” Accessibility of IMM channels to membrane impermeant inhibitors such as toxins indicates that the extracellular domains of the channels face the IMS. Such is the case of, i.e., K_V_1.3 [21], BK (K_Ca_1.1) [22], and ASIC1a [23], whereas for other mitochondrial channels, the topology has not been established. 

Like any other membrane potential in biology, Δψ_m_ is determined by ionic gradients and changes when ions flow in and out across the IMM. In turn, reactive oxygen species (ROS) production, which depends on the efficiency of electron transport, is modulated by the activity of IMM channels [24], but can also change the activity of these channels [25]. Moreover, the function of mitochondria as Ca^2+^ stores is dependent on both Δψ_m_ and [Ca^2+^]_i_ (or rather the Ca^2+^ flow at mitochondrial contact sites with ER and lysosomes, see below) [26]. Besides driving activation of the mitochondrial permeability transition pore (mPTP), the Ca^2+^ concentration in the matrix controls the permeation through (at least) the multiple types of Ca^2+^ activated K^+^ (K_Ca_) channels present at the IMM [20,27,28] (Section 2.3). The ion fluxes across the IMM also impact mitochondrial volume [29] and mitochondrial dynamics [30], both important for mitochondrial function, as further detailed below. Finally, in addition to being Ca^2+^-regulated, both the mPTP [31] and several of the IMM channels are very sensitive to pH (e.g., TASK3 [32,33], BK [34,35], and ASICs [36]), further emphasizing the interconnectedness of these parameters. 

Thus, whereas the literature on ions in mitochondrial function—and vice versa, the importance of mitochondria in regulating cytosolic ion homeostasis—has almost exclusively focused on Ca^2+^, this relationship cannot be studied in isolation, as changes in cytosolic and mitochondrial Ca^2+^ are interdependent with changes in H^+^ and K^+^ concentrations in determining mitochondrial function (Figure 2B). For example, in mammalian cells, an increase in [Ca^2+^]_i_, such as after stimulation of cells with histamine elicits a transient cytosolic acidification, at least in part due to H^+^ counter-transport by Ca^2+^-extruding ATPases. This alters the driving force for H^+^, translating to a transient decrease in the pH of the mitochondrial matrix. Notably, the mitochondrial acidification induced by such stimuli can be pronounced, exceeding by far the cytosolic acidification due to the much lower H^+^ buffering capacity of the matrix [37]. At the same time, however, stimulation with Ca^2+^-mobilizing agonists elicits a Ca^2+^-induced increase in respiration which favors matrix alkalinization. Thus, the net effect of an increase in [Ca^2+^]_i_ on mitochondrial pH depends on the relative magnitudes of these opposite effects. Similarly, while cytosolic K^+^ concentrations change much less than those of Ca^2+^ and H^+^, the importance of K^+^ in setting Δψ_m_, and the co- or counter-transport of K^+^, H^+^, and Ca^2+^ by many of the mitochondrial coupled transporters, makes changes in all of these ions highly interdependent. This interdependence is important to keep in mind as we briefly outline the key roles of each ion below. 

### 2.1. Ca^2+^ in Mitochondrial Function

[Ca^2+^]_i_ needs to be maintained in the range of 10^−7^ M [38,39], and mitochondria are a crucial organelle for that purpose [40], while also an early victim of Ca^2+^ homeostasis alterations [26,41]. Several enzymes of the tricarboxylic acid (TCA) cycle are Ca^2+^ dependent, and therefore the efficiency of ATP synthesis also depends on the Ca^2+^ concentration in the matrix [42]. Hence, matrix Ca^2+^ influences ROS production among other mechanisms by modifying the efficiency of respiration [43]; ROS modulate ion channels at the IMM, whose activity in turn can change Δψ_m_, and thereby the driving force for Ca^2+^ flux and for ETC function, altering both matrix Ca^2+^ and ROS production [44,45] (Figure 2B). 

The fact that mitochondria can store relatively high amounts of Ca^2+^ has been known for many years. Part of that Ca^2+^ is present as insoluble salts [46], but the peak free Ca^2+^ concentration in the matrix can reach hundreds of µM during signaling [47] (Figure 2A). 

**Figure 3:**
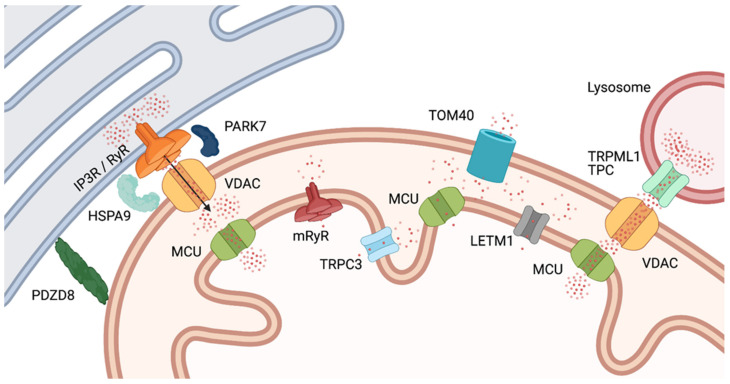
Calcium gradients at MAM microdomains. At ER-mitochondria (**right**) and lysosome-mitochondria (**left**) contacts, Ca^2+^ concentration reaches levels that allow efficient import through MCU. The ER MAM provides colocalization between the Ca^2+^ release receptor (through HSPA9 and PARK-7) and VDAC1. Ca^2+^ (represented by red dots) flowing through the receptor will thus enter the IMS readily. Lysosome-mitochondria contacts serve a similar purpose, the Ca^2+^ source being in this case TRPML7 channels. At other locations, IMS Ca^2+^ will reach lower concentrations, and transport through MCU will be less efficient. For abbreviations and definitions, see text.

**Figure 4:**
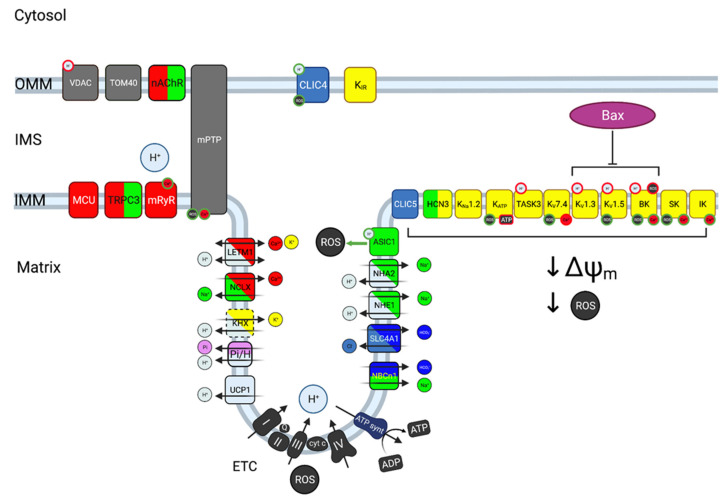
Cartoon representing some of the ion transport proteins detected at mitochondria. The relevant ions are color-coded (K^+^ yellow, Na^+^ green, H^+^ light blue, Ca^2+^ red, Cl^−^ blue, HCO_3_^−^, dark blue, P_i_^−^ pink). Non-selective channels are represented in the colors of the ions they permeate. For the case of transporters (indicated by arrows), the substrates of exchangers are indicated by the corresponding colors, in parallel for cotransporters and diagonal for exchangers. The dashed line for KHX denotes its disputed identity. Regulating factors are represented by circles on the transport protein, with a green border for activators and a red border for inhibitors. Not all transporters will be represented in a given cell, and some important transporters (e.g., aquaporins) have been omitted.

The first barrier to the entry of Ca^2+^ into the mitochondria is the OMM. As noted above, the high ion permeability of the OMM is no longer regarded as “passive”: the voltage-dependent anion channel (VDAC), which makes large pore-forming structures permeable to multiple metabolites and ions including Ca^2+^, dominates the permeability of the OMM [48], but it is not the only channel in that location. In yeast, several transporters allow ion flow in addition to their primary cargo, and two OMM ion channels besides VDAC have been reported [49]. Higher eukaryotic mitochondria also express at least an inward rectifier K^+^ channel [50], nicotinic acetylcholine receptors [51] and CLIC4, a member of the intracellular Cl^−^ channel family [52], in the OMM (Figure 4). Although the translocon channel TOM40 (the pathway for cellular proteins entering the mitochondria) is also permeable to cations, high cytosolic Ca^2+^ concentrations are best achieved at microdomains in the proximity of Ca^2+^ entry or -release channels. VDAC proximity and interaction with such channels at ER-mitochondrial contacts (Figure 3) makes it a key component of regulated Ca^2+^ entry [48]. Ca^2+^ entry through VDAC is a regulated process that seems to occur during low-conductance gated states rather than together with the full opening of the channel to large anions [53]. Molecular dynamic simulations indicate that these transitions are controlled by changes in the intrinsically disordered N-terminus of the channel [54]. 

Ca^2+^ entry occurs primarily at mitochondria-ER contact sites known as mitochondria-associated membranes (MAMs). At the MAMs, inositol-trisphosphate receptor (IP3R) channels interact through Glucose-regulated protein 75 (a member of the HSP70 family, HSP9A) [55] and DJ-1 (a.k.a. the Parkinsonism-associated deglycase, PARK-7) [56] with VDAC in the OMM, resulting in a high [Ca^2+^] microdomain in the particular region of the IMS [57] (Figure 3). Mitochondria-ER tethering through the ER protein PDZD8 is also required for proper Ca^2+^ uptake in the mitochondria [58]. The high local Ca^2+^ concentration created in this manner is essential because, once the ion has passed through the OMM, the main route for Ca^2+^ entry into the matrix is the mitochondrial calcium uniporter (MCU) in the IMM, which shows low Ca^2+^ affinity. Despite its name, the MCU is a channel [59]. The µM Ca^2+^ concentration required for efficient Ca^2+^ transport by MCU is rarely found in the cytoplasm per se, but occurs at the MAMs. Structurally, the MCU is a protein complex, with MCU1 forming the channel, which is activated by MICU1 and inhibited by MICU2. MICU1 and -2 form a heterodimer that controls MCU1 activity. MCU activity is very low at basal Ca^2+^, rises steeply in response to a Ca^2+^ increase, and decays at high Ca^2+^ concentrations to prevent Ca^2+^ overload. MCU Ca^2+^ dependence may be conferred by an additional member of the complex, Essential MCU Regulator (EMRE). EMRE^−/−^ mice lack rapid mitochondrial Ca^2+^ uptake but are viable and resistant to mPTP activation [26]. 

Other pathways for Ca^2+^ entry into the matrix, albeit less extensively characterized, are mitochondrial ryanodine receptors (mRyR, [60,61], transient receptor potential canonical type 3 (TRPC3) channels [62], and possibly Leucine-zipper and EF-hand-containing transmembrane protein 1 (LETM1) (see Section 2.2). A fourth rapid Ca^2+^ uptake mechanism (RaM) with unknown molecular substrate operates at low Ca^2+^ concentrations and could correspond to a different mode of operation of MCU since MCU knockout abolishes this pathway (reviewed in [63,64]) (Figure 3). 

Thus, while a signal activating the IP3R in the ER will increase bulk [Ca^2+^]_i_, the first structure outside of the ER that senses the increase will be the mitochondria, and the concentration entering the IMS through VDAC will probably (transiently) be more than enough to promote transport through the MCU. Hence, changes in [Ca^2+^]_i_ are not only mirrored but to some extent preceded by changes in mitochondrial matrix Ca^2+^, which will drastically modify mitochondrial physiology. The process requires a coordinated action of IP3R, VDAC, and MCU, and its specific properties will therefore depend on tissue and cell type [65]. 

In addition to the ER, also lysosomes form direct contact sites with mitochondria which regulate mitochondrial dynamics. Similar to the MAMs, mitochondria-lysosomal contact sites colocalize with fission sites [66]. These contacts, which are at least in part dependent on Rab7 on the lysosomes [66], are also sites of inter-organelle Ca^2+^-signaling, as the lysosomal cation channel TRPML1 was recently shown to mediate Ca^2+^ transfer to the mitochondrial matrix via VDAC and MCU [67], i.e., a mechanism rather analogous to that involving IP3R-mediated Ca^2+^ transfer to the matrix from the ER. Moreover, lysosomal two-pore channels (TPCs) might be involved in this process [68] (Figure 3). 

The inflow of Ca^2+^ into mitochondrial needs to be exquisitely regulated, as excessive or prolonged increases in mitochondrial Ca^2+^ can activate the mPTP, triggering mitochondrial swelling, Δψ_m_ collapse, bioenergetic failure, and eventually necrotic cell death (e.g., [69], see Section 3.1). It is therefore not surprising that mitochondrial Ca^2+^ uptake is regulated at all levels, from the release at MAMs to MCU. For example, it was recently reported that local ROS signaling controls IP3R activity at the MAM and, thereby, reduces Ca^2+^ entry, resulting in protection against cell death [70]. 

### 2.2. Protons in Mitochondrial Function

As drivers of ATP production, H^+^ ions are at the center of mitochondrial metabolism. The first estimates of the pH gradient across the IMM were obtained in isolated rat liver mitochondria by Mitchell and Moyle in 1969 [71], followed by measurements in the 1970s by Nichols [72] and Rottenberg [73]. These measurements arrived at a matrix pH of between 7.5 and 8.2 at an outer pH of 7.0. Later measurements in various intact cells using pH-sensitive dyes [74], cardiomyocytes, or mitochondria-targeted genetically encoded pH sensor probes [75], HeLa cells and rat cardiomyocytes; [76], HeLa cells and rat cortical neurons; [37], HeLa cells) determined matrix pH values of 7.6–8.2 under resting conditions, apparently similar between normal and cancer cells. Importantly, the pH of the IMS was determined at 6.88 in human ECV304 cells [77], i.e., slightly more acidic than the cytosol, despite the high ion permeability of the OMM (Figure 2A), confirming that different ion concentrations can indeed arise between the cytosol and the IMS. 

Multiple H^+^ transporters are found in the IMM (Figure 4). The respiratory complexes I, III and IV mediate the transport of H^+^ from the matrix to the IMS driven by the ETC, rendering the matrix alkaline and the IMS acidic [78]. In addition to its contribution to the Δp driving the ATP synthase, the H^+^ gradient generated in this manner drives import of inorganic phosphate for ATP synthesis via a Pi-H^+^ cotransport system of the SLC25 family, and electroneutral import of Ca^2+^, Na^+^, and K^+^ across the IMM, via H^+^ exchange with Ca^2+^, Na^+^, and K^+^ (for a review, see [79]). While this transporter has been a topic of controversy, the mitochondrial Ca^2+^/H^+^ exchanger is most probably encoded by LETM1 [80,81]. LETM1 appears to directly sense both H^+^ and Ca^2+^ [82] and to play transport-unrelated roles in signaling, including its physical anchoring to the mitochondrial ribosome protein L36 [83]. The nature of the mitochondrial Na^+^/H^+^ exchanger (NHE) is also controversial. Mitochondrial localization of SLC9A6 (NHE6) was postulated but later disputed [84]. SLC9A1 (NHE1), which predominantly localizes to the plasma membrane, was detected in cardiomyocyte mitochondrial fractions [85], and also the related transporter SLC9B2 (NHA2) appears mitochondrially localized in some cells [86,87]. The molecular identity of the mitochondrial K^+^/H^+^ exchange mechanism remains highly contentious, and it remains possible that also this function involves LETM1 [81,88]. The mitochondrial uncoupling protein UCP1 is also a H^+^ channel, albeit this isoform is specific to brown adipose tissue [89], and of course also the highly permeable mPTP carries H^+^. 

To our knowledge, essentially nothing is known about the possible roles of bicarbonate transporters in mitochondrial pH homeostasis. This is of interest given their key roles in cytosolic pH regulation and the fact that mitochondria also harbor the carbonic anhydrase activity required for conversion of metabolically produced CO_2_ to HCO_3_^−^ [90]. Consistent with the existence of bicarbonate transporters in this organelle, mitochondrial localization of a protein related to the anion exchanger SLC4A1 (AE1) [91], and more recently, the electroneutral Na^+^,HCO_3_^−^ cotransporter SLC4A7 (NBCn1) [92] has been reported. Supporting the notion that CO_2_/HCO_3_^−^ is important for mitochondrial function independent of its effect on pH, the HCO_3_^−^-regulated soluble adenylate cyclase (sAC) is expressed in mitochondria [93]. Here, sAC is activated by HCO_3_^−^ downstream from metabolic CO_2_ production, resulting in local cAMP production, and activation of protein kinase A (PKA) in the mitochondrial matrix. This in turn regulates OXPHOS through phosphorylation of several ETC proteins [93]. Emphasizing the interdependence between ionic signaling processes, sAC is also activated by Ca^2+^ [94].

The difference between the slightly acidic IMS and the alkaline matrix contributes to the electrochemical driving force for H^+^, also known as the proton-motive force (Δp), across the IMM. Δp, which is of fundamental importance for life as the driver of mitochondrial ATP production, is the sum of the chemical difference in H^+^ concentration across the membrane, and Δψ_m_. While Δψ_m_ provides most of Δp, the H^+^ gradient between the acidic IMS and the alkaline matrix contributes with about 20–30% of Δp and is independently important through its contribution to metabolic substrate transport and mitochondrial ion homeostasis [79]. Beyond these key roles in driving ATP synthesis, matrix pH regulates several other mitochondrial processes including Ca^2+^-signaling, apoptosis, and at least indirectly also the fission/fusion balance, as further discussed in subsequent sections. In principle, H^+^ transport between the matrix and the IMS could result in cytosolic pH changes given the high permeability of the OMM. However, given the low H^+^ buffering capacity of the matrix compared to the cytosol [37], such a contribution would likely be relatively minor. 

### 2.3. Potassium in Mitochondrial Function

The K^+^ concentration is only slightly higher in the mitochondrial matrix (150–180 mM [14,15]) than in the cytosol and therefore in the IMS (100–120 mM), but the magnitude of Δψm provides a strong driving force for K^+^ into the matrix (Figure 2A). K^+^ flow into the matrix serves two main functions: In addition to modulating Δψ_m_, K^+^ plays a crucial role in controlling mitochondrial volume, which affects the local concentration of all matrix components, including Ca^2+^, H^+^, nucleotides and other metabolites [14,95]. Consistent with these important regulatory functions, the IMM displays a large variety of different K^+^ channels (Figure 4). In most cases, their functions are mainly inferred from the properties of the corresponding channels at the plasma membrane. We know that they are present at the IMM and that their inhibition, overexpression or knockdown alters mitochondrial physiology, but the specific roles in this organelle remain, with some exceptions, mostly hypothetical. The presence of a K^+^ channel inhibited by ATP in the IMM was first inferred by the protective effects that a K^+^ permeation pathway with similar pharmacological properties to the plasma membrane K_ATP_ channel showed in ischemia-reperfusion models [14,96]. The channel was found to result from the association of K_IR_1.1 (ROMK [97]) and a mitochondrial sulfonylurea receptor encoded by a splice variant of SUR2 [98]. The mitochondrial channel would respond to matrix ATP content and, accordingly, modulate Δψ_m_. Its overexpression causes mitochondrial swelling, suggesting that the channel participates in the control of mitochondrial volume and its adaptation to the ATP needs of the cell [96]. Exogenous ROS is able to activate the mitochondrial K_ATP_ [25]. K_Ca_ channels are also present in the IMM. Members of the small conductance (SK) family exert a protective role in ischemia-reperfusion models in guinea pig [99]. The intermediate conductance (IK) K_Ca_ channel (K_Ca_3.1) is also present at the IMM in some cell types, where it would contribute to K^+^ conductance upon increases in Ca^2+^ in the matrix [100,101]. Probably the best characterized IMM K_Ca_ channel is the large conductance (BK) channel K_Ca_1.1. Like that of its lower conductance counterparts, activation of this channel appears cytoprotective [20,102]. Mitochondrial BK activity will depend on both Δψ_m_ and Ca^2+^, as the Ca^2+^ sensitivity of BK channels is voltage-dependent [103]. Notably, its functions may extend beyond ion transport, or at least involve close proximity with other transporters: BK is physically (through the accessory subunit β4) and functionally coupled to the respiratory chain, and could modulate the activation of mPTP [20]. BK is inhibited by external acidification [34], while internal H^+^ have the opposite effect [35]. Thus, all families of K_Ca_ channels are present at the IMM, and their different Ca^2+^- (and for BK also voltage-) dependence provide the mitochondria with a tool to control matrix volume and Δψ_m_ as a function of Ca^2+^. Some of the members of the K_Ca_ family are in turn modulated by ROS [104]. A Na^+^-activated K^+^ channel (K_Na_1.2) has also been identified in the IMM; cells from K_Na_1.2 knockout mice showed a deficit in adapting to high energy demand when only fat was the energy source [105]. 

Due to the large voltage gradient across this membrane, the presence of voltage-gated K^+^ (K_V_) channels at the IMM is not intuitively logical, since the potential for semi-maximal activation of these channels is in the range between −40 and 0 mV [106]. At least K_V_1.3 [21], K_V_1.5 [107], and K_V_7.4 [108] have been identified in the IMM, as has TASK3 [109] (Figure 4). This K^+^ channel is strongly inhibited by external H^+^, establishing a direct link between H^+^ in the IMS and K^+^ conductance [32,33]. 

All the K^+^ channels present at the IMM have a plasma membrane counterpart. It is however not clear if these proteins are structurally and functionally identical at both locations. The fact that the function of channels in this superfamily depends on both specific location and chemical environment (e.g., the presence of phosphatidylinositol (4,5) bisphosphate (PI(4,5)P_2_) stabilizes the open state of K_V_7.4) [106] and association with other proteins or subunits (for example, the above-mentioned interaction of BK with the ETC relies on the accessory subunit β4) [20] would point to specific features and behavior of channels at the IMM, but further studies are needed to unravel these. 

## 3. Mitochondrial Architecture, Dynamics, and Morphology in Normal Cells

Multiple processes integral to normal cell function and cell fate are associated with regulation of mitochondrial architecture, dynamics, and morphology [110]. These will be briefly introduced below, followed by a discussion of the molecular mechanisms involved and their links to ion transport (Section 3.2). This will provide the necessary background for a discussion of their interplay with ion transport in cancer (Section 4). 

### 3.1. Physiological Processes Dependent on Changes in Mitochondrial Architecture

Reduced nutrient availability frequently favors mitochondrial fusion over fission resulting in a hyperfused mitochondrial network. This not only increases mitochondrial ATP production capacity [111], but also protects mitochondria against mitophagy [112], because this process is dependent on mitochondrial fragmentation. Mitophagy—the process of selective degradation of depolarized or damaged mitochondria, is driven by mitochondrial quality control mechanisms and protects cells against deleterious effects of damaged mitochondria, such as excessive ROS levels [113,114]. Induction of autophagy *per se* causes mitochondrial hyperfusion, triggered by PKA-mediated phosphorylation of the fission protein DRP1 (Section 3.2) and likely serving to prevent unintended mitochondrial degradation [115]. In another protective mechanism, p53-dependent, transient fusion of mitochondria with lysosomes induces C-terminal cleavage of VDAC and marks mitochondria to escape autophagy [116]. This process is linked to cellular Ca^2+^ levels, as cleavage can occur through asparagine endopeptidase or the Ca^2+^-dependent protease calpain 1 [117]. Mitochondrial hyperfusion also occurs in response to other stressors such as oxidative stress and extracellular acidosis (further discussed in Section 5). In this context, it is often referred to as stress-induced mitochondrial hyperfusion (SIMH), and again likely serves as a protective measure, which increases OXPHOS efficiency and limits cell death. Moreover, cristae morphology is altered by such stressors, and this is important for tuning OXPHOS efficiency to the cellular requirements [118]. 

During cell cycle progression, mitochondrial morphology undergoes extensive, cyclic reorganization to ensure correct distribution between the daughter cells [119]. For instance, during G1-S transition, mitochondrial morphology was shown to change from isolated, fragmented elements into a hyperfused, giant network. Prevention of this change blocked cell cycle progression, while conversely, experimentally inducing mitochondrial hyperfusion caused quiescent cells to begin replicating their DNA and build up expression of cyclin E (the cyclin responsible for G1 to S transition) [120]. Thus, mitochondrial reorganization is an integral part of the control of cell cycle progression. Interestingly, mitochondrial architecture, or more specifically, tethering of mitochondria to the plasma membrane, is important for spindle organization in budding yeast [121,122], where distribution of mitochondria between mother and bud is a highly regulated process. In higher eukaryotes, where mitochondria are randomly distributed among daughter cells, fragmented mitochondria migrate to the division furrow immediately before cytokinesis in a process dominated by plasma membrane-mitochondrial contacts [123]. 

Also the balance between stemness and differentiation is intricately related to mitochondrial dynamics, and, notably, this appears to be tightly linked to the interplay between mitochondria and Ca^2+^ signaling. Thus, stem cell self-renewal potential and differentiation have been found to be dependent on mitochondrial (hyper)fusion in a manner involving regulation of Ca^2+^—Notch interplay driven by mitochondrial fusion [124], and regulation of ROS levels by mitochondrial dynamics [125,126]. 

Cell motility is increasingly recognized to be dependent on major mitochondrial rearrangement. In *Drosophila melanogaster* embryos, mitochondria dynamics determine the efficacy of wound healing by controlling both Ca^2+^ signaling and actin dynamics, and lack of Drp1 results in failure in epithelial repair [127]. Moreover, in mammalian cells, migration requires intense membrane trafficking, cytoskeletal reorganization and cyclic release of focal adhesions and generation of new ones, all processes using ATP that result in the need of local enrichment in energy production [128,129]. In directionally migrating cells, mitochondria are asymmetrically distributed and enriched in the leading edge, and this requires both proper mitochondrial dynamics and preserved interaction with microtubules [130]. In general, mitochondria are reportedly fragmented at the leading edge cortical zone, which would allow them to occupy the narrow spaces inside filopodia and lamellipodia. Interestingly, mitochondria inside filopodia show clear MAMs (suggesting active local Ca^2+^ signaling) and exhibit a dense matrix in electron microscopy, indicative of activation [131]. 

Cell death in essentially all its forms is causally linked to changes in mitochondrial dynamics and morphology [132]. Necrosis—and at least in some cell types also its regulated form, necroptosis—involves opening of the mPTP—a high-conductance, channel that spans the OMM and IMM and allows permeation of molecules up to a molecular weight of 1.5 kD. The molecular identity of mPTP remains contentious and multiple candidates have been brought forward (reviewed in [31]). Opening of the mPTP dissipates Δψ_m_ and causes mitochondrial swelling, eventually leading to rupture [133]). Interestingly, Ca^2+^ and H^+^ exert opposite effects on mPTP activity: Ca^2+^ potently activates it, while acidic pH inhibits it, a phenomenon that has been widely studied in the context of cardiac ischemia [31].

During apoptosis, the proapoptotic Bcl-2 proteins Bax and Bak translocate to mitochondria, forming a pore in the OMM, in a manner that remains to be fully understood. This mitochondrial outer membrane permeabilization (MOMP) leads to release of cytochrome c and other pro-apoptotic IMS molecules, including SMAC/Diablo, enabling apoptosome formation and caspase activation [132,134]. Strikingly, MOMP is also associated with a macropore formation that allows release of mitochondrial DNA (mtDNA) despite its localization not in the IMS but in the mitochondrial matrix. This results in a type I interferon response, as the presence of mtDNA in the cytosol activates the cyclic GMP-AMP synthase (cGAS)-stimulator of interferon genes (STING) pathway that otherwise recognizes foreign double-stranded DNA as part of the innate immune system [132]. 

Mitochondria undergo massive DRP1-dependent fragmentation during early stages of apoptosis, and this is necessary for subsequent cell death, possibly by facilitating cytochrome c release through cristae reorganization [132,134]. Importantly however, extensive fission can also interfere with OMM permeabilization [135]. Finally, less studied forms of cell death, including paraptosis and ferroptosis, also involve mitochondrial reorganization [132]. As discussed in the following section, all of these death processes are closely linked with changes in ionic flux. 

Finally, it is increasingly appreciated that mitochondria are key cellular signaling platforms, and that also this is tightly dependent on mitochondrial architecture [136]. While it seems likely that many mitochondrial signaling processes are dependent on mitochondrial ion transport, this question is so far essentially only explored for ROS, which we will therefore focus on there. While ROS production is dependent on mitochondrial architecture, the relation is not linear, as high levels of ETC activity, inhibition of ETC activity, and damaged mitochondria not removed by mitophagy, can all give rise to mitochondrially derived ROS [114,137]. ROS signaling is not only closely related to Ca^2+^ homeostasis [44], but also impacts mitochondrial ion homeostasis by regulating mitochondrial ion channels and transporters [138]. This will be discussed further in Section 5.5. 

### 3.2. Regulation of Mitochondrial Architecture: A Highly Ca^2+^-Dependent Process

The shape, size and position of the mitochondrial network result from an equilibrium between biogenesis, mitophagy, fusion, fission, transport, anchoring, and deformation of the mitochondria [3], a dynamic process that can result in morphological changes in a timescale of seconds [139]. The basic concepts of regulation of mitochondrial architecture, morphology and dynamics have been reviewed extensively [3,140]. Briefly, the key proteins responsible for mitochondrial fission and fusion belong to the class of dynamin-related proteins. In mitochondrial fusion, these comprise the mitofusins, MFN1 and -2, located in the OMM, and OPA1, located in the IMM. Together with the mitochondrial contact site and cristae organizing system (MICOS), OPA1 is also important for cristae formation and stability [140]. Mitochondrial fission is dependent on another dynamin-related protein, DRP1, which translocates from the cytosol to the OMM, where it binds its OMM partners MID49, MID51, MFF, and FIS1. Mitochondrial fission occurs at the MAMs, where interactions between the ER-localized formin INF2 and its mitochondrial binding partner induce actin polymerization and actomyosin-dependent mitochondrial deformation. As outlined above (Section 2.1), Ca^2+^ released from the ER through the IP3R channel at MAMs (or released from lysosomes through TRPML1 or TPC) enters the mitochondrial matrix passing first through VDAC in the OMM, and next via MCU in the IMM. This causes IMM fission, followed by complete mitochondrial fission as DRP1 oligomerizes around the OMM [3]. A detailed account of the signaling pathways involved in these changes is beyond the scope of this review, but it is important to point out that they are often Ca^2+^ dependent. For instance, PKA-dependent phosphorylation of Ser637 of DRP1 inhibits DRP1 GTPase activity, leading to hyperfusion through inhibition of fission, while Ca^2+^-dependent dephosphorylation of the same site by calcineurin is associated with mitochondrial fragmentation and cell death [141]. Conversely, mitochondrial fission and fusion influence Ca^2+^ signaling in the mitochondria. Thus, acute inhibition of fission caused increased MCU activity and enhanced mitochondrial Ca^2+^ uptake in mouse muscle in vivo [142] and in cell culture, where inhibition of fusion had the opposite effect [143].

Finally, also mitochondrial trafficking along the microtubule network is Ca^2+^-dependent. This process involves the Ca^2+^-dependent Rho-like GTPases Miro1 and −2 and their binding partners TRAK1 and −2, which link mitochondria to kinesin microtubule motor proteins (reviewed in [110,144]. Upon increases in [Ca^2+^]i, kinesin is released from Miro-TRAK and instead binds to syntaphilin (SNPH). SNPH inhibits the kinesin, causing the mitochondria to be immobilized [145]. 

### 3.3. Impact of H^+^ and K^+^ Homeostasis on Mitochondrial Architecture—and Vice Versa

Given the above-described interrelationship between Ca^2+^, H^+^, and K^+^ fluxes in key mitochondrial functions, and the widely studied role of Ca^2+^ in regulation of mitochondrial architecture it is surprising that H^+^ and K^+^ are generally largely ignored in this context. This likely in part reflects technical limitations (mitochondria-localized, genetically encoded reporters for Ca^2+^ are much more widely used and characterized than corresponding reporters for other ions for instance). It could also be related to the misconception that of these ions, only Ca^2+^ is a signaling molecule. Yet it is by now recognized that H^+^ also serves as a bona fide signaling molecule [146], and in the case of K^+^, the pivotal role of this ion in regulating Ca^2+^ flux, and setting Δψ_m_ and volume, warrants analysis of its impact on mitochondrial architecture. Despite this partial knowledge gap, there are multiple indications that also H^+^ and K^+^ regulate mitochondrial architecture and hence function, under physiological conditions. For instance, mitochondrial hyperfusion is induced by extracellular acidosis [147]. There is also a causal link between caspase activation during apoptosis and mitochondrial pH homeostasis, as caspase activation is promoted by mitochondrial alkalinization and concomitant cytosolic acidification caused by reverse-mode transport via the ATP synthase [148]. Furthermore, it has been suggested that cristae remodeling between condensed and orthodox conformation is pH dependent [149].

Interestingly, mitochondrial dynamics can also have striking effects on mitochondrial pH. Thus, OPA1 activity was shown to elicit flashes of matrix alkalinization that propagated between continuous mitochondria. These pH flashes, the nature of which remains incompletely understood, were proposed to be energy conservation events involved in equilibration of Δψ_m_ after fusion events [150].

As described in Section 2.3, K^+^ flow plays fundamental roles in mitochondrial volume regulation [16,29]. In ischemia/reperfusion models, K^+^ influx triggers anomalous fusion of fragmented mitochondria and formation of toroid mitochondria [151]. In dopaminergic neurons treated with rotenone to induce mitochondrial fragmentation, block of K_ATP_ channels diminished fragmentation, while activation of the channel increased it, although it is unclear whether this effect relies on the IMM channel population [30]. Plasma membrane K^+^ channels have also been reported to influence mitochondrial dynamics, by still unclear mechanisms that could involve changes in Ca^2+^ homeostasis [152]. 

## 4. General Features of Mitochondrial Architecture in Cancer Cells

Studies on mitochondrial morphology in patient tumor tissues are scarce, and often focus on the ultrastructure of the mitochondria rather than on the structure of the network per se [153]. Although it is difficult to establish a general rule in this respect, tumor cell mitochondria often appear more fragmented than those of normal cells. This in part reflects oncogenic signaling directly—for instance, Ras [154] and Myc [155] both drive marked changes in mitochondrial architecture. However, in addition to this, the profound cellular changes associated with transformation and the harsh, and spatiotemporally variable, microenvironmental conditions in tumors also impact mitochondrial morphology, and may, as discussed below, also result in the opposite phenomenon—mitochondrial hyperfusion. Thus, a wide variety of mitochondrial architectures can be expected in cancer cells in tumors. 

An example of a link between fragmented mitochondria and cancer was reported in mouse and human ovarian cancer cell lines, in which mitochondria changed with increasing malignancy from a filamentous network to single, rounded structures localized more closely adjacent to the nucleus, due to an imbalance of fusion and fission regulators. These changes aided the adaptation to hypoxia through the promotion of autophagy and were accompanied by changes in the mitochondrial ultrastructure, Δψ_m_, and ROS levels [156]. Similarly, in human invasive breast carcinoma, mitochondria were more fragmented in metastatic than in nonmetastatic cells, due to upregulation of DRP1 and downregulation of MFN1. Silencing DRP1 or overexpressing MFN1 caused mitochondrial elongation or clustering, inhibited lamellipodia formation, and suppressed metastatic capacity and chemoattractant-induced recruitment of mitochondria to lamellipodial regions [157]. 

## 5. Cancer Cell Functions Driven by Changes in Mitochondrial Architecture

By far the majority of studies of mitochondria in cancer have taken a metabolic perspective. Here, we will focus on how the physiological processes of mitochondrial reorganization and—signaling—all of which are, as described above, highly dependent on ion transport between the cytosol and the mitochondrial compartments—are exploited by cancer cells to drive aggressive cell behaviors. Specifically, we will discuss how this may be causally linked with the dysregulated ionic homeostasis in cancer cells. While not further elaborated here, it is important to note that not only the cancer cells, but also other cells in the tumor microenvironment, including immune cells and fibroblasts, exhibit changes in mitochondrial dynamics, sometimes in an interplay with those observed in the tumor cells (e.g., [158], reviewed in [159]). 

### 5.1. Survival in the Harsh Tumor Microenvironment

As noted above, nutrient limitations, hypoxia, oxidative stress, and profound extracellular acidosis are key properties of the tumor microenvironment [6,160], and similar to normal cells, tumor cells respond to such stressors with mitochondrial hyperfusion. One of the first and still most thorough analyses of this phenomenon [161] showed that when nutrient availability was switched from glucose to galactose/glutamine, HeLa cells developed a hyperfused mitochondrial network that allowed them to derive energy by glutaminolysis. Compared to those of cells growing in a glucose-rich medium, these hyperfused mitochondria were thinner, more oxidized, and had higher expression of ETC proteins. Their matrix morphology changed from orthodox to condensed configuration, they had more cristae, and their matrix pH was substantially less alkaline, presumably reflecting the use of the H^+^ gradient to drive import of metabolites and inorganic phosphate [161]. Thus, it is worth noting that the fragmented nature of mitochondria reported in tumor cells cultured in growth medium at pH 7.4 and 21% O_2_, may not be generally representative of cancer cells within the tumor microenvironment. The advantage for the cancer cells likely goes beyond this metabolic flexibility, as hyperfusion is protective through inhibition of mitophagy in cancer ([114]; Section 3.1).

### 5.2. Cell Proliferation, Stemness, and Senescence

Increasing evidence suggests that the importance of mitochondrial architecture in cell cycle progression as described in Section 4 may represent a potential target in cancer. A study in MCF7 and MDA-MB-231 human breast cancer cells showed that DRP1 inhibition delayed G2-M progression and elicited aneuploidy, due to replication stress as a consequence of the mitochondrial hyperfusion and untimely build-up of cyclin E in the G2 phase [162]. Similarly, the DRP1/MFN2 ratio was increased in lung cancer patient tumor tissue, and DRP1 knockdown or inhibition blocked cell cycle progression in lung cancer cells [163]. In mouse models of PDAC, induction of mitochondrial fusion or inhibition of fusion, either through pharmacological or genetic manipulations, reduced tumor growth [164]. 

Moreover, cancer stem cell properties appear dependent on both fusion, fission, and mitophagy to maintain appropriate ROS levels (for a review, see [165]). Accordingly, DRP1 inhibition inhibited mammosphere forming capacity and stemness-related signaling in breast cancer cells [166]. Linking mitochondrial reorganization driven by ionic dysregulation, extracellular acidic stress induces mitochondrial hyperfusion [147], and acidosis was shown to promote and maintain glioma stem cell properties in a manner involving increasing mitochondrial respiration and ATP production [167]

Furthermore, both oncogene- and radiation-induced senescence is associated with increased mitochondrial biogenesis, fusion, and reduced mitophagy [168]. The mechanistic link, if any, is incompletely understood but seems likely to involve increased ROS levels. While further evidence is needed, we speculate that inducing senescence is another way in which altered mitochondrial dynamics could play an important role in cancer cells, because senescent cells are protected from chemotherapy-induced death, yet can contribute to cancer development through the senescence-associated secretory phenotype (SASP) (for a review, see [169]). 

### 5.3. Cell Migration and Invasion

DRP1 was shown to be upregulated in invasive breast carcinomas, lymph node metastases, and cell lines prone to metastasis, and reducing fission (by silencing DRP1 or overexpressing MFN1) inhibited metastasis formation [157]. Similarly, in hepatocellular carcinoma, DRP1 is more abundant in metastases while MFN1 is more abundant in the primary tumor. DRP1 would promote migration in this case through facilitating loosening of focal adhesions via canonical Ca^2+^/CaM/ERK/FAK signaling, and therefore in a Ca^2+^-dependent fashion [170]. In glioblastoma, NF-κB inducing kinase (NIK) promotes mitochondria fission and increases migration through direct modulation of DRP1 [171]. In most cases, therefore, fission seems to favor cell migration. In contrast, elongated mitochondria were reported at the cortical area of glioblastoma cells where migration was stimulated by PI3K inhibitors, while MFN1 silencing blocked the effect [172]. A genome-wide shRNA screening of genes whose knockdown was able to rescue the invasive phenotype after treatment with a mitochondria-targeted HSP90 inhibitor identified SNPH (Section 3.2), as a major factor implicated in invasion. In tumor cells, SNPH reduces the speed and distance that mitochondria travel along microtubules. At the same time, SNPH would modulate mitochondrial dynamics [173]. Recent studies showed that in addition to regulating mitochondrial localization and motility, SNPH also regulated mitochondrial ROS production, and identified SNPH as a key stress-regulated switch between proliferation and migration-invasion in tumors [174]. The dependence on in some cases fission and in others apparently fusion could be an expression of context-dependence, as genetic and environmental factors can influence the way mitochondria respond to the needs of tumor cells under different circumstances [4]. Still, it could also be part of the same phenotypic adaptation, as elongated mitochondria travel faster than fragmented ones [173]. Finally, it is notable that the process of epithelial to mesenchymal transition (EMT), which is generally considered important for the increased migratory capacity of tumor cells, induces mitochondrial fusion [126].

The role of ion fluxes in migrating cells has been extensively studied (e.g., [175]). Specifically, Ca^2+^ at the leading edge of migrating cells plays a pivotal role by controlling both adhesion and protrusion/retraction cycles of lamellipodia [176]. Given the crucial roles of mitochondria in modulating Ca^2+^ signals [26], it seems likely that the role of locally abundant mitochondria is not limited to providing the required energy for migration. Specially in the restricted space of the leading edge, the rapid Ca^2+^ uptake by the mitochondria, followed by slower release, can help shape the amplitude and duration of Ca^2+^ oscillations [177] and, thereby, modulate migration.

Collectively, these data highlight that the requirement for regulated changes in mitochondrial architecture in the process of migration is not a simple matter of either fusion or fission, but involves both processes, likely in a context-dependent manner. 

### 5.4. ROS Production and Signaling

ROS levels are often increased in cancer cells and contribute to multiple aspects of tumorigenesis and metastasis, from DNA damage causing genetic instability, to growth, angiogenesis, metastasis, and apoptosis resistance [137,178]. Yet, while it is clear that sublethal levels of ROS can be pro-tumorigenic, antioxidants have been shown to favor tumor growth, and the possible relevance of ROS as a target of anticancer treatment is contentious (see e.g., [179,180]). The increased levels of mitochondrially derived ROS in cancer cells likely stem from a combination of factors, including both increased production and reduced free radical scavenging [137]. Importantly, there is a close, reciprocal interplay between Ca^2+^ homeostasis and ROS, which has been assigned important roles in cancer biology [44]. Mitochondrial pH is inherently linked to ROS production through the ETC. However, it is also interesting to note that several studies reporting mitochondrial ROS flashes have likely been studying mitochondrial pH flashes (Section 3.3), given the stronger sensitivity of the reporters to pH than to ROS [181]. 

Mitochondrial ion homeostasis is regulated by ROS signaling through direct and indirect regulation of numerous ion channels and transporters [138]. In addition to the K^+^ channels discussed above, this includes also the IP3R and RyR [138]. The link is not limited to the mitochondrial proteins, as ROS play important yet incompletely understood roles in regulation of plasma membrane-localized channels and transporters implicated in cancer. For instance, mitochondrial fragmentation was shown to elicit ROS-dependent inhibition of Na^+^/H^+^ exchangers both in mammalian cells and *C. elegans* [182]. It is also interesting to note that adaptation of cancer cells to growth in the acidic tumor microenvironment profoundly rewires their redox biology [183]. Whether this is related to the shift toward mitochondrial respiration under these conditions [7] is yet unknown, but it again points to the reciprocal regulatory interactions between cellular and mitochondrial ion homeostasis. 

### 5.5. Resisting Cell Death

A large-scale genetic and pharmacological screen demonstrated that interfering with either fission or fusion made cancer cells hypersensitive to apoptosis induced by SMAC mimetics [184]. In ovarian cancer, hypoxia-induced mitochondrial fission correlated with cisplatin resistance [185]. This was suggested to involve ROS signaling, as hypoxia increased ROS levels, ROS treatment similarly increased mitochondrial fission, and DRP1 inhibition sensitized cells to cisplatin chemotherapy [185]. On the other hand, increased mitochondrial biogenesis (mediated by peroxisome proliferator-activated receptor-gamma coactivator (PGC)-1α) also promoted resistance to cisplatin [186], and, as noted above, mitochondrial hyperfusion (SIMH) appears to be an adaptive pro-survival response, accompanied by increased mitochondrial ATP production. Notably, SIMH can precede mitochondrial fission triggered by apoptotic stimuli such as UV irradiation or actinomycin D [111], presumably reflecting an initial attempt by the cell to repair damage, followed by death when the stress is excessive. Whereas the roles of mitochondria in apoptotic and necrotic cell death have been by far most widely studied, they also play key functions in other forms of cell death [132]. One such pathway is paraptosis, in which ER and mitochondria dilate in absence of classical features of apoptosis and necrosis [187]. The process is induced by a number of natural compounds and anticancer drugs [188], including compounds previously considered specific NHE1 inhibitors [189] and is of interest in the treatment of cancer cells, which have downregulated apoptotic machinery. Notably, while the mechanisms involved in paraptosis are still incompletely understood, substantial evidence links it to Ca^2+^ dysregulation, and also BK channel dysfunction and disturbed K^+^ homeostasis has been suggested [188]. 

K_V_1.3 participates in programmed cell death acting downstream of Bax and Bak [21]. High doses of mitochondria-targeted K_V_1.3 inhibitors induce apoptosis, while sublethal concentration increase proliferation [19], indicating a regulatory role of the channel in the process. Upon specific mitochondrial K_V_1.3 inhibition, a transient mitochondrial hyperpolarization followed by cytochrome c release and depolarization precede cell death [21]. Inhibition of K_V_1.5, possibly through similar mechanisms, also induces apoptosis [190]. It is unclear, however, if this concept can be extended to other K^+^ channels, although Bax had no effect on K_Ca_3.1 (IK) channels [101]. 

## 6. Dysregulation of Mitochondrial Ion Channels and Transporters in Cancer

Similar to mitochondrial metabolic enzymes, mitochondrial ion channels and transporters are often dysregulated in cancer and several hold potential as interesting for anticancer therapy [191]. Ca^2+^ signaling plays a relevant role in most if not all tumor-relevant cellular processes [127]. Altered Ca^2+^ uptake in mitochondria has been detected in many cancer types, and often a role in tumor development has been proposed. VDAC overexpression or mutation has similarly been reported in many cancer types [192]. The interaction of VDAC with hexokinase II that favors a metabolic switch towards glycolysis and protects against apoptosis has been extensively studied [193]), but the evidence that altered Ca^2+^ uptake through VDAC is mechanistically linked to its pro-tumorigenic role is mainly indirect, and relies on the importance of Ca^2+^ for ETC, ROS production, and apoptosis [45,193,194].

Consistent with the complex biology of MCU (Section 2.1), both pro- and anti-tumorigenic roles of MCU have been reported in cancers. Thus, MCU was overrepresented in more aggressive and metastatic breast cancers, and downregulation of MCU in metastatic cell lines resulted in impaired growth, migration, and invasion in vitro, and metastasis and lymph node infiltration in vivo. This was interpreted to be a result of loss of HIF-1α adaptive signaling in response to increased ROS production upon Ca^2+^ uptake [195]. MCU (or MCUR1) activity was also found to be prometastatic in hepatocellular carcinoma [196,197,198]. On the other hand, MCU is a target for miR-25, which is highly expressed in cancers and cancer cell lines, and downregulation of MCU through miR-25 increases resistance to apoptosis in PC3 and HCT116 cells [199]. In pancreatic cancer, uncontrolled activation of MCU by histidine triad nucleotide binding-2 (HINT2, a mitochondrial adenosine phosphoramidase) resulted in enhanced apoptosis and increased sensitivity to gemcitabine [200]. It is thus possible that the action of MCU is context-dependent: tumors may benefit from enhanced MCU expression in advanced stages, while in early stages MCU can impair tumor progression.

TRPC3 is an alternative Ca^2+^ entry pathway through the IMM [62] (Section 2.1). This channel has been shown to play relevant roles in the development of different tumor entities [201], prominently in ovarian [202,203] and triple negative breast cancers [204], but the implication of mitochondrial TRPC3 in these cells has not yet been demonstrated.

LETM1 expression is increased in many cancers [83,205]. In most cases, LETM1 appears to be pro-tumorigenic [206,207]. The open questions surrounding the substrate specificity and stoichiometry of LETM1 (Section 2.2) preclude understanding of roles of LETM1 mediated transport in cancer. If LETM1 serves as an electroneutral 1Ca^2+^/2H^+^ exchanger, the driving forces across the IMM in energized mitochondria would favor Ca^2+^ efflux via this transporter (Figure 2). In this case, it might be speculated that LETM1 upregulation can protect cancer cells from mitochondrial Ca^2+^ overload (whereas it might exacerbate such overload if the driving force changes, i.e., in depolarized mitochondria). Complicating the matter, the importance of LETM1 in tumors may not be limited to ion transport. For instance, LETM1 overexpression in HeLa cells induced a metabolic shift leading to ATP depletion and necrosis, apparently in a manner related to its interaction with mitochondrial ribosome protein L36 [83].

Beyond LETM1, the extent to which the mitochondrial acid/base transporters might contribute specifically to cancer development has, to our knowledge, not been directly studied. Numerous studies have, however, established the importance of specific acid/base transporters in essentially all aspects of cancer development, growth and metastasis. In this regard it is notable that NHE1 and NBCn1, some of the acid/base transporters most widely shown to be upregulated in cancer cells and play key roles in cancer development [8,9,208,209,210], have both been found to localize to mitochondria. Given the pivotal importance of mitochondrial pH and bicarbonate in regulation of both OXPHOS (Section 2.2) and of cancer-associated mitochondrial metabolic events, including the recently discovered pH-dependent production of the oncometabolite l-2-hydroxyglutarate (l-2HG; [211]), it will be important to unravel the roles of specific mitochondrial acid/base transport changes in a cancer setting.

Similar to the net acid-extruding transporters, the activity of mitochondrial K^+^ channels is in general related to cytoprotection, and their inhibition facilitates cell death (Section 2.3). Most studies addressing these channels are related to ischemia-reperfusion, hypoxia or stroke, all situations where protecting cells from death is therapeutically desirable. As a general rule, a correlation between the abundance of a particular channel in the plasma membrane and in the IMM is expected [28]. Since many of the K^+^ channels detected at the IMM belong to the long list of channels overexpressed in tumor tissues [11], it is reasonable to hypothesize that K^+^ channels will be more abundant in many cancer cells, but this needs experimental corroboration. Channels like TASK3, BK, and IK play very relevant roles in cancer pathology, but whether this is relevant also for their mitochondrial versions remains to be investigated. IK inhibition resulted in enhanced apoptosis in the presence of TRAIL in melanoma [212], while TASK3 activation rendered reduced viability in glioma [213]; again, the extent to which this can be attributed specifically to the mitochondrially localized channels is unknown. K_V_1.3, however, stands out from this general scenario. Non-permeant K_V_1.3 blockers (i.e., blocking only plasma membrane-localized channels) reduce cell proliferation, but do not induce cell death. In contrast, permeable agents (or those targeted to the mitochondria), induce apoptosis. Inhibition of mitoK_V_1.3 was first shown to be required for apoptotic cell death in T-cells and macrophages. Blockade of the channel by Bax, by a mechanism analogous to that of known K_V_1.3 inhibitory peptide toxins, triggers apoptosis by inducing Δψ_m_ depolarization, ROS accumulation and cytochrome c release. This sequence of events is mimicked by addition of non-permeant blockers to isolated mitochondria, which can be considered unequivocal evidence for the participation of mitochondrial K_V_1.3 and not plasma membrane K_V_1.3 [21].

A similar mechanism of direct blockade by Bax could apply for K_V_1.5. Intriguingly, overexpression of K_V_1.1 specifically in mitochondria restored apoptosis in lymphocytes lacking both K_V_1.3 and K_V_1.5, suggesting a common mechanism shared by the K_V_1 channel family [190]. Bax was also shown to directly inhibit the mitochondrial BK and induce opening of the mPTP [214]. However, because plasma membrane BK channels can participate in apoptotic cell shrinkage in glioma cells upon activation of the extrinsic apoptosis pathway, it is difficult to discern the exact participation of the mitochondrial channel in intact cells [215,216,217]. Therefore, researchers have designed mitochondria-targeted K_V_1.3 blockers that successfully induced apoptosis in explanted tumor cells from chronic B cell leukemia and in animal models of melanoma and PDAC [218]. Repurposed cell-permeant K_V_1.3 blockers were also efficacious in orthotopic models of PDAC [219].

## 7. Conclusions and Perspectives

Once regarded as a mere power source for the cell, mitochondria have in recent years moved towards the center of cellular signaling and cell physiology. In the context of cancer, the metabolic functions of mitochondria are both shaped and exploited by the cancer cells, allowing them to deal with the challenges of the tumor microenvironment, including nutrient shortage, low O_2_, and acidic extracellular pH. As we have discussed in this review, such metabolic adaptation already represents and significant advantage for cancer cells, but it only one of multiple potential effects of the mitochondrial changes that favor cancer progression. Because mitochondria act as both reservoir and modulator of Ca^2+^ signaling, changes in mitochondrial Ca^2+^ handling can affect all processes that rely on Ca^2+^ signaling, from cell cycle and secretion to cytoskeletal reorganization. Moreover, ROS, predominantly produced in an ion transport-dependent manner in mitochondria, exert profound pro-metastatic changes in cell behavior. Both mitochondrial H^+^ transport and K^+^ transport drive mitochondrial changes that regulate key hallmarks of cell metabolism and fate, in turn impacting cancer development. Finally, essentially all forms of cell death rely on mitochondria, rendering this organelle key to the sensitivity of cancer cells to death-inducing factors, both endogenous and exogenous, such as chemotherapy.

These adaptations are driven by oncogenic transformation as well as by selection in the harsh microenvironmental conditions in the tumor. They involve both biochemical and architectural changes and likely an increased plasticity, allowing the mitochondrial network to adapt to the particular needs of the cell in the spatially and temporally heterogenous tumor microenvironment. A common denominator of those adaptations are changes in mitochondrial ion transport and, reciprocally tied to this, changes in the ionic gradients across the mitochondrial membranes. As we have described, these gradients not only define the energy state of the organelle, but also have crucial roles in mitochondrial fission/fusion balance, volume, and signaling, as well as in mitochondrial positioning through cytoskeletal interactions, a process important for cell cycle progression and cell motility.

Many of the ion channels and transporters responsible for controlling those ionic gradients have been identified in recent years, providing insight into the complexity of the system. Unexpectedly, a vast majority of these proteins were already known for their important roles at the plasma membrane or other intracellular membranes. In many cases, their properties are indistinguishable from those of corresponding channels and transporters at other locations, while the consequences of their function are radically different. A critical future question will be the posttranslational regulation of these mitochondrial channels and transporters, which is, with a few exceptions, largely enigmatic today.

We have limited our scope to Ca^2+^, H^+^, and K^+^ ion gradients, but investigation into the mitochondrial transport of other ions will certainly reveal further complexity in the future. For example, mitochondria contain 20–50% of cellular iron and serve as cellular iron stores. Mitochondrial iron uptake is essential for heme synthesis and iron-sulfur cluster biogenesis [220,221]. The mechanism of iron transport through the OMM remains incompletely understood, while the best studied mechanism of iron transport through the IMM involves mitoferrin-1 (MFRN1, SLC25A37) [220]. Cancers are highly iron dependent, at least in part through the roles of iron in mitochondrial metabolism, however, high iron levels are also associated with altered redox signaling and ferroptosis, and it has been suggested that this may constitute a therapeutic opportunity [222]. Magnesium, the most obvious importance of which lies in its requirement to form Mg-ATP, is imported through the IMM by MRS2 and accumulated in mitochondria, which then act as a store modulating its cytoplasmic concentration [223]. Copper is required for the catalytical function of cytochrome c oxidase and superoxide dismutase [224], and is transported through the IMM by the phosphate transporter SLC25A3 [225]. Finally, zinc enters the mitochondria via SLC39A1 (ZIP1) and MCU; among other roles, the level of zinc in the matrix participates in quality surveillance during fission events [226].

The extensive genetic heterogeneity of cancer cells even within a single tumor, and the spatiotemporal heterogeneity of the tumor microenvironment, in conjunction with the strong adaptive ability of cancer cells, complicates the process of translating understanding of mitochondrial ion transport to therapeutic opportunities. Nevertheless, the dependence of mitochondrial function upon ion gradients indicates that therapeutic approaches could be directed to compensate for the advantages that tumor cells gain from such molecules. A particularly interesting group are the channels implicated in resistance to apoptosis. K_V_1.3 is a chief example in this regard since, as discussed, only the population residing in mitochondria exerts protection against apoptosis. Thus, mitochondria-targeted blockers could eliminate the resistance to apoptosis of some tumor types, and we hypothesize that a similar scenario could be the case for K_V_1.5. For some channels, such as BK channel, blockade of the mitochondrial channel is expected to be pro-apoptotic while blocking the plasma membrane channel will, at least in some cases, be antiapoptotic, making such a strategy challenging. In any case, a better understanding of the differences between ion channels and transporters in mitochondria and their plasma membrane counterparts is likely to unravel a potential to exploit such differences for therapy.

Mitochondrial function and architecture are fundamentally dependent on the concentrations of, and driving forces for, key cellular ions. Conversely, mitochondrial function impacts cellular ion homeostasis. Thus, mitochondrial function and its cellular consequences cannot be understood without knowledge of ion transport, and without taking the interdependency between cytosolic and mitochondrial ion concentrations into account. While complicating the understanding of both mitochondrial changes and ion transport in cancer, this interdependence also provides new opportunities for therapeutically targeting these changes.

## Abbreviations

BK, large (big) conductance Ca^2+^-dependent K^+^ channel (*KCNMA1*, K_Ca_1.1); [Ca^2+^]_i_, the free cytosolic Ca^2+^ concentration; CLIC, Intracellular Chloride Channel; Δp, the proton-motive force; Δψm, mitochondrial membrane potential; DJ-1, parkinsonism-associated deglycase, PARK-7; EMRE, Essential MCU Regulator; EMT, epithelia-to-mesenchymal transition; ETC, electron transport chain; FIS1, mitochondrial fission 1 protein; GRP75, Glucose-Regulated Protein 75; HINT2, histidine triad nucleotide binding-2; IK, intermediate conductance Ca^2+^-dependent K^+^ channel (*KCNN4*, K_Ca_3.1); IMM, inner mitochondrial membrane; IMS, intermembrane space; INF2, Inverted Formin 2; Kv, Voltage-gated potassium channel (*KCNA*); l-2HG, l-2-hydrogyglutarate; LETM1, Leucine-zipper and EF-hand-containing transmembrane protein 1; MAM, mitochondria-associated membrane; MCU, mitochondrial Ca^2+^ uniporter; MFF, mitochondrial fission factor; MID49/51, mitochondrial dynamics protein of 49/51 kDa; Miro1/2, Mitochondrial Rho 1/2; MFN, mitofusin; MICOS, mitochondrial contact site and cristae organizing system; MMP, mitochondrial membrane permeabilization; MOMP, mitochondrial outer membrane permeabilization; mPTP, mitochondrial permeability transition pore; OMM, outer mitochondrial membrane; OPA1, optic atrophy 1; PKA, protein kinase A; ROS, reactive oxygen species; sAC, soluble adenylate cyclase; SASP, senescence-associated secretory phenotype; SIMH, stress-induced mitochondrial hyperfusion; SK, small conductance Ca^2+^-dependent K^+^ channel (*KCNN2*, K_Ca_2.2) SNPH, Syntaphilin; TASK3, Twik-related acid-sensitive K^+^ channel (*KCNK9*, K_2P_9.1); TCA, Tricarboxylic acid cycle; TOM40, translocase of the outer membrane-40; TRAK1, Trafficking Kinesin; TRPC, Transient Receptor Potential, Canonical type; TRPML, Transient Receptor Potential, mucolipin type; VDAC, Voltage-dependent Anion channel.
